# Optimized Pepper Target SNP-Seq Applied in Population Structure and Genetic Diversity Analysis of 496 Pepper (*Capsicum* spp.) Lines

**DOI:** 10.3390/genes15020214

**Published:** 2024-02-07

**Authors:** Yihao Wang, Xiaofen Zhang, Jingjing Yang, Bin Chen, Jian Zhang, Wenyue Li, Heshan Du, Sansheng Geng

**Affiliations:** 1 State Key Laboratory of Vegetable Biobreeding, Beijing Vegetable Research Center, Beijing Academy of Agriculture and Forestry Science, Beijing 100097, China; wangyihao@nercv.org (Y.W.); chenbin@nercv.org (B.C.); 2 National Engineering Research Center for Vegetables, Beijing Vegetable Research Center, Beijing Academy of Agriculture and Forestry Science, Beijing 100097, China; zhangxiaofen@nercv.org (X.Z.); yangjingjing@nercv.org (J.Y.); zhangjian@nercv.org (J.Z.); 3 Beijing Key Laboratory of Vegetable Germplasms Improvement, Beijing 100097, China; 4 Key Laboratory of Biology and Genetics Improvement of Horticultural Crops (North China), Beijing 100097, China; 5 Henan OULAND Seed Industry Co., Ltd., Zhengzhou 450003, China; 13592613788@139.com

**Keywords:** pepper, target SNP-seq, fruit shape, genetic diversity, population structure

## Abstract

Peppers are a major vegetable crop worldwide. With the completion of additional genome assemblies, a multitude of single-nucleotide polymorphisms (SNPs) can be utilized for population structure and genetic diversity analysis. In this study, we used target SNP-sequencing as a new high-throughput sequencing technology, screening out 425 perfect SNPs for analyzing the genetic diversity and population structure among 496 pepper lines from five pepper species in China and abroad. The perfect SNP panel exhibited commendable discriminative ability, as indicated by the average values of polymorphism information content, observed heterozygosity, minor allele frequency, and genetic diversity, which were 0.346, 0.011, 0.371, and 0.449, respectively. Based on phylogenetic, population structure, and principal component analyses, 484 *C. annuum* lines were divided into four subpopulations according to the shape of fruit: blocky fruit, wide-horn fruit, narrow-horn fruit, and linear fruit. These subpopulations displayed clear clustering with minimal or no overlap. Moreover, F statistic (*Fst*) analysis revealed considerable distinctions among these subpopulations. Additionally, we established a set of 47 core SNPs that could effectively differentiate among all pepper lines. This core SNP set could precisely classify the *C. annuum* lines into four distinct fruit-shape groups. The blocky and narrow-horn fruit subpopulations displayed the lowest and highest genetic diversity, respectively. This study highlights the importance of fruit shape as a crucial trait in pepper breeding. Moreover, this work indicates the immense potential of optimized target SNP technology in the addition of foreground markers of important traits to improve molecular breeding efficiency, and demonstrates its broad application prospects in the genetic analysis and variety identification of peppers.

## 1. Introduction

Pepper (*Capsicum* spp.) is an important vegetable crop that originated in South America and is cultivated worldwide [1]. According to the Food and Agriculture Organization, global pepper cultivation reached 2.05 million hectares in 2021, resulting in a total production of 36.29 million tons [2]. China is the largest pepper-producing country in the world, with a cultivation area of 0.76 million hectares and a production of 16.75 million tons [2]. A total of 42 species of pepper have been reported to date, including five domesticated species: *Capsicum annuum* L., *Capsicum frutescens* L., *Capsicum chinense* Jacq., *Capsicum baccatum* L., and *Capsicum pubescens* Ruiz and Pav. [3]. Among them, *C. annuum* is the most widely cultivated domesticated species worldwide. Continuous selection and breeding efforts have contributed to the development of pepper varieties with diverse fruit shapes, colors, and flavors [4].

Over the past few decades, with the continuous expansion of the seed market, a growing number of commercial pepper varieties have emerged. The identification and certification of varieties have become increasingly important to protecting the interests of breeders and producers. Traditionally, variety identification was achieved via morphological characteristics determined in field investigations; however, this approach is time-consuming, labor-intensive, and susceptible to environmental interference [5]. The use of molecular markers and the establishment of fingerprints as an assisting method provide more convenience and efficiency for variety identification and DUS testing and are better suited to meet current high-throughput detection requirements [6]. With completion of the whole-genome sequences and assemblies of multiple pepper varieties, including Zunla-1, CM334, and Ca59 [1,7,8], many simple sequence repeat (SSR) and insertion–deletion (InDel) markers have been developed and utilized to detect pepper genetic diversity and domestication traits, as well as for variety identification [9,10,11,12,13]. However, SSR and InDel markers, along with random amplified polymorphic DNA and restriction fragment length polymorphism markers, have limitations and cannot be widely used for identification on a large scale [14,15]. Alternatively, single-nucleotide polymorphisms (SNPs) are the most abundant and genetically stable variations in the genome, which offer several advantages as markers, including rapid and accurate high-throughput detection and easy integration of genotype information, making them ideal for analyzing genetic backgrounds [16,17].

SNP-based genotyping techniques such as cleaved amplified polymorphic sequences (CAPS) and derived CAPS have been extensively utilized in genetic research [18,19]. However, these methods have certain limitations owing to the inability of electrophoretic systems to meet the current demands of rapid and large-scale testing. With the continuous advancement of high-throughput sequencing technologies, SNP genotyping platforms have emerged, including competitive allele-specific polymerase chain reaction (KASP) and DNA microarrays [20,21]. Nevertheless, these methods are relatively expensive, and their usage is constrained by time-consuming design-related detection procedures, thereby limiting their suitability for genotyping a substantial number of SNPs and samples [22]. Currently, genotyping by target sequencing technology is widely employed as an SNP genotyping method that integrates multiplex PCR and next-generation sequencing technologies, which has also been successfully commercialized and applied to various crops [23,24,25,26,27].

In contrast to KASP, microarray, whole-genome resequencing, and other SNP genotyping methods, SNP sequencing (SNP-seq) is a more effective and cost-efficient method. SNP-seq offer flexibility in terms of the number of SNPs and samples used, while demonstrating high genotyping accuracy for highly polymorphic and conserved sequences within the genome [28]. In particular, target SNP-seq technology combines multiplex amplification with high-throughput sequencing. A higher sequencing depth (with an average depth of 1000×) ensures a higher SNP detection rate and accuracy in genotyping, thereby reducing the occurrence of false-positives and false-negatives. This technology was first applied to the genetic analysis and variety identification of 261 cucumber varieties [6]. Target SNP-seq technology has since been successfully employed to construct fingerprints in crops such as cucumber [28], melon [29], eggplant [30], watermelon [31], and pepper [32].

In our previous study, we used target SNP-seq technology to analyze the genetic diversity of 271 commercial pepper varieties based on 92 perfect SNPs [32]. However, there were a limited number of SNPs for this analysis, and the collected pepper varieties were restricted to commercial hybrids. Therefore, in the present study, we optimized target SNP-seq technology by selecting 425 perfect SNP loci and adding several important trait loci, significantly enriching the density and quality of markers. In addition, we collected 496 pepper lines from five pepper species in China and abroad. Genetic diversity and population structure analyses of these pepper lines based on SNP markers provide valuable insights into artificial selection in pepper breeding. The selected core-SNPs set, consisting of 47 SNP markers, can also be used for pepper variety identification and intellectual property protection. Adding important foreground markers in the SNP panel can improve the efficiency of pepper molecular breeding.

## 2. Materials and Methods

### 2.1. Plant Material and DNA Isolation

A total of 496 pepper lines were included in this study (Table S1): 69 were obtained from the U.S. National Plant Germplasm System, 115 were provided by Henan Ouland Seed Industry Co., Ltd. (Zhengzhou, China), 222 were sourced from the pepper genetic breeding group of Beijing Vegetable Research Center (BVRC), and 90 were introduced from the Vegetables Germplasm System of Beijing Academy of Agriculture and Forestry Sciences. Additional information regarding these pepper lines is provided in Table S1. DNA was extracted from the fresh leaves of seedlings of each inbred line using the modified CTAB method [33], and DNA quality was determined by 1.5% agarose gel electrophoresis.

### 2.2. Resequencing and Genome-Wide SNP Discovery

Genome-wide SNP discovery was performed using resequencing data from 31 pepper inbred lines that were previously sequenced by the Pepper Genetic Breeding Group of BVRC (publicly accessible at http://bigd.big.ac.cn/gsa accessed on 8 August 2023, accession number CRA001576) [32], along with reported sequencing data from four cultivars: Dempsey, Zunla-1, Perennial, and Chiltepin. All high-quality reads were aligned to the reference genome Zunla-1 v2.0 [1] using the Burrows–Wheeler alignment (BWA) tool with default parameters, and the mapped reads were filtered out by PCR duplication. Subsequently, perfect SNPs were selected from the whole genome of pepper using Genome Analysis Toolkit (GATK, v2.4-7g5e89f01) [34], with the following stringent filtering criteria: (1) minor allele frequency (MAF) > 0.4; (2) missing rate < 0.2; (3) heterozygosity < 0.2; (4) no sequence variations in the flanking region of 100 bp; and (5) two alleles per SNP locus.

### 2.3. SNP Genotype Analysis by Target SNP-Seq

Library construction for target SNP-seq was performed according to the target SSR-seq protocol reported previously [6], which involved two rounds of PCR. At first, a multiplex PCR panel was used with primers targeting the 150-bp flanking regions of the perfect SNPs. In the second round, a unique barcode was introduced to the amplified products of each sample to enable their differentiation. The purified PCR products from each sample were then combined to prepare the target SNP-seq library, which was subsequently subjected to sequencing on an Illumina HiSeq X Ten platform (Molbreeding Biotechnology Company, Shijiazhuang, China).

The Illumina bcl2fastq pipeline (Illumina, San Diego, CA, USA) was employed to demultiplex the raw data obtained from target SNP-seq. This pipeline uses a sample-unique barcode to determine the precise genotype of each inbred line. Next, the clean data were filtered using Trimmomatic, and the reads of each inbred line were aligned to the reference genome of pepper, Zunla-1 v2.0, using BWA with default parameters. SNP genotypes were subsequently named using GATK. Based on the high-throughput sequencing results, alleles with the highest and second-highest read counts were defined as the major and minor alleles, respectively. A locus was identified as homozygous if the read frequency of the major allele exceeded 0.7. If both the major and minor allele read frequencies were >0.35, the locus was classified as heterozygous.

### 2.4. Genetic Structure Analysis of Pepper Lines

Three methods, namely, principal component analysis (PCA), population structure, and phylogenetic tree construction, were used to analyze the genetic relationships among pepper lines. STRUCTURE v2.3 [35], which is based on a Bayesian model approach, was employed to analyze the population structure following standard procedures. Twenty independent runs were conducted with the cluster number (K) ranging from 1 to 15, a burn-in period of 100,000 iterations, and 100,000 Monte Carlo Markov Chain simulations. The K value was determined using Evanno’s delta K method [36].

PCA was performed using the FactoMineR package in R [37]. An unrooted phylogenetic tree was constructed using the neighbor-joining method with the Ape and Poppr packages in R [38] and was further enhanced using iTOL for improved visualization [39].

### 2.5. Population Diversity Analysis of Pepper Lines

Perl script was used to calculate the MAF, genetic diversity (GD), observed heterozygosity (Ho), and polymorphism information content (PIC) of subpopulations with different fruit shapes [6]. We performed AMOVA within and between groups [40], as well as pairwise Fst analysis [41] using the poppr and Hierfstat R packages, respectively, to determine the genetic differences between populations and subpopulations.

### 2.6. Selection of the Core-SNPs Set for Inbred Line Discrimination

A Perl script was used to analyze the genetic diversity at each SNP site to screen the minimum number of SNPs required to distinguish the maximum number of pepper lines [6]. The selected SNPs were recognized as the core-SNP set with high discrimination. After obtaining the core-SNP set, saturation curves were generated by conducting pairwise comparisons of the genotypes for all pepper lines.

### 2.7. Selection of Core Pepper Lines

Within each subpopulation, a pairwise comparison matrix was constructed by counting the number of differential SNP genotypes between each pair of pepper lines and considering missing genotypes as null values [6,42]. A lower number of differential SNP genotypes for a given pepper line compared with the others indicated a closer relationship. The top 10% of pepper lines with a closer relationship than the others in each subpopulation were considered the core pepper lines for this analysis.

## 3. Results

### 3.1. Genome-Wide Perfect SNPs Identified

We successfully mapped our previously sequenced resequencing data of 31 inbred pepper lines [32] to the reference genome Zunla-1 v2.0 [1]. We also identified raw polymorphic SNPs across the entire genome sequences of these 31 inbred pepper lines and four cultivars with available full-genome sequences: Dempsey, Zunla-1, Perennial, and Chiltepin [7]. Considering the large number of highly repetitive sequences in the pepper genome, we refined the selection of candidate SNPs for target SNP genotyping.

**Figure 1:**
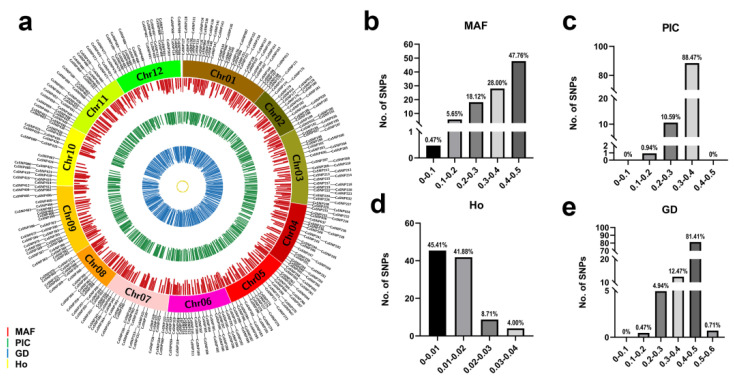
Genetic characteristics of 425 perfect single-nucleotide polymorphisms (SNPs) in 496 pepper lines. (**a**) Distribution of 425 SNP loci on the 12 pepper chromosomes. (**b**) Minor allele frequency (MAF). (**c**) Polymorphic information content (PIC). (**d**) Observed heterozygosity (Ho). (**e**) Genetic diversity (GD).

A total of 425 perfect SNPs were selected for target SNP genotyping and were evenly distributed across the 12 pepper chromosomes, with an average of 35 SNPs on each chromosome. The average physical distance between SNP markers was 5.92 Mb (Figure 1a; Tables S2 and S3).

### 3.2. Genotyping of Pepper Lines Using Target SNP-seq

A total of 496 pepper lines were genotyped with the 425 perfect SNP loci using target SNP-seq. Additionally, fingerprints for each of the pepper lines were established using these 425 perfect SNPs (Table S4). Overall, 47.76% of these perfect SNPs exhibited an MAF between 0.4 and 0.5, suggesting a better discriminating ability (Figure 1b). The PIC values of the perfect SNPs ranged from 0.100 to 0.384, with a mean of 0.346. Furthermore, 81.41% of the SNPs showed a PIC greater than 0.3, while only four SNPs showed the PIC less than 0.2 (Figure 1c). The Ho values for each SNP ranged from 0 to 0.038, with a mean of 0.011, indicating that the 496 pepper lines had high homozygosity (Figure 1d). The average GD of the 425 SNPs was 0.449, ranging from 0.106 to 0.504 (Figure 1e). Notably, three SNPs exhibited an inbreeding coefficient of 1, indicating the absence of heterozygous genotypes in all pepper lines (Table S4). Overall, these results revealed that the 425 perfect SNPs of pepper possess high discrimination power and are suitable for genetic diversity analysis.

### 3.3. Genetic Structure of Pepper Lines

**Figure 2:**
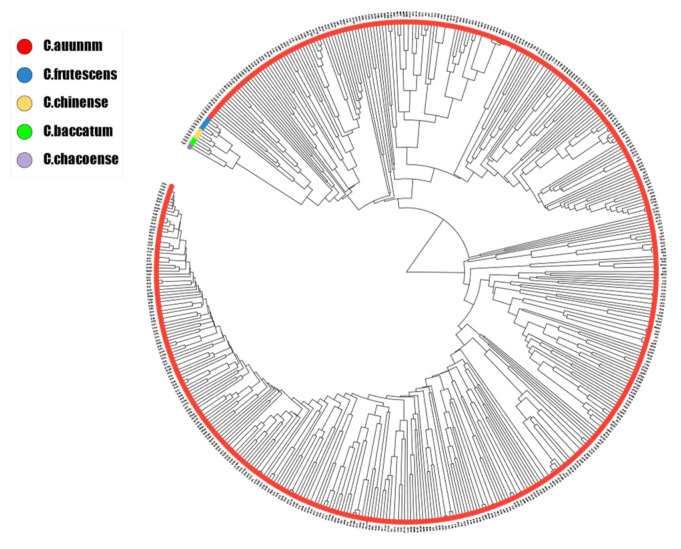
Neighbor-joining tree of 496 pepper lines based on 425 perfect single-nucleotide polymorphisms.

The genetic structure of the 496 pepper lines was analyzed based on the 425 perfect SNPs selected above. Most of these pepper lines were bred in China, with only a small proportion imported from abroad. A phylogenetic neighbor-joining tree was constructed, and the genetic distances were well-arranged according to interspecific relationships. Most of the pepper lines belonged to *C. annuum*, with three lines each belonging to *C. baccatum* and *C. frutescens*, one line belonging to *C. chacoense*, and five lines belonging to *C. chinense* (Figure 2; Table S1).

**Figure 3:**
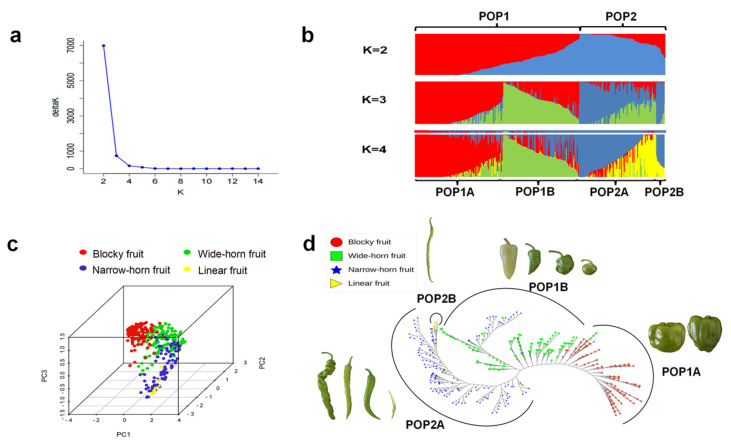
Population structure analysis of 484 *C. annuum* lines. (**a**) Delta K value derived from target SNP-seq genotype data using STRUCTURE. (**b**) All *C. annuum* lines were divided into two main populations (POP1 and POP2) when K = 2, and four subpopulations (POP1A, POP1B, POP2A, and POP2B) when K = 4. (**c**) Principal component analysis (PCA) of 484 *C. annuum* lines. (**d**) Unrooted neighbor-joining tree of the four subpopulations.

To further analyze the genetic structure of the 484 *C. annuum* lines, the most likely number of clusters (K-value) was determined to be two, suggesting the presence of two main populations (Figure 3a), which were designated POP1, comprising 314 pepper lines (64.9%), and POP2, comprising 170 pepper lines (35.1%). Increasing the K-value to three revealed that POP1 could be further divided into two subpopulations based on fruit shape: POP1A, which predominantly consisted of blocky fruit pepper lines, nine wide-horn fruit lines, and one narrow-horn fruit line; and POP1B, comprising mostly wide-horn fruit lines and a small percentage of narrow-horn fruit lines. When the K-value was set to four, POP2 was further divided into two subpopulations: POP2A, dominated by narrow-horn fruit lines with a few linear fruit lines and one wide-horn fruit line (similar to POP1); and POP2B, comprising only 11 linear fruit lines. Overall, the four subpopulations (POP1A, POP1B, POP2A, and POP2B) exhibited a clear structure with minimal mixing, primarily driven by different fruit shapes (Figure 3b; Table S5).

PCA was conducted to investigate the clusters of the 484 *C. annuum* lines using the 425 perfect SNPs. The PCA results were consistent with those of the analyses described above, demonstrating that the four populations clustered separately according to different fruit shapes. The blocky and wide-horn fruit lines exhibited more concentrated clustering, whereas the narrow-horn and linear fruit lines exhibited greater dispersal along the PCA plot. Interestingly, the linear and blocky fruit lines did not overlap in the cluster, indicating higher diversity and more pronounced genetic differentiation. Notably, the narrow-horn and linear fruit populations appeared to be more closely related despite their distinguishable fruit shapes (Figure 3c).

The unrooted neighbor-joining tree of the *C. annuum* lines constructed based on pairwise genetic distances provided additional support for the PCA- and model-based population structure analyses, revealing obvious differences among the four populations in terms of fruit shape, although some mixtures were observed between the wide- and narrow-horn fruit lines (Figure 3d). The representative lines corresponding to the fruit shapes of the subpopulations are depicted in Figure 3d. Collectively, the division of *C. annuum* lines into four genetic populations associated with different fruit shapes was strongly supported by three independent analyses. This suggests that fruit shape has played a significant role in the selection and breeding of these *C. annuum* lines.

### 3.4. Identification of a Core-SNP Set

**Figure 4:**
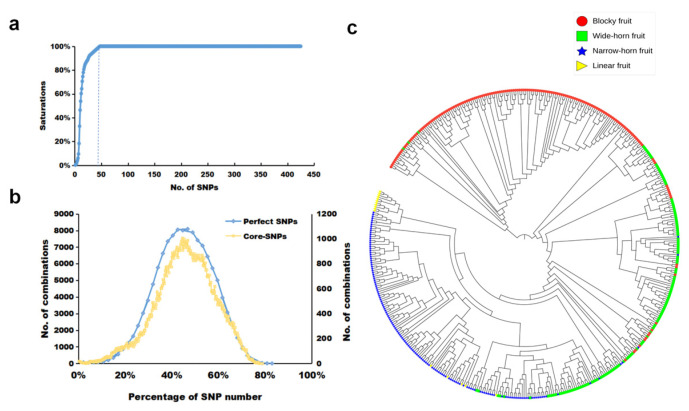
Identification ability of 47 core single-nucleotide polymorphisms (SNPs) in 496 pepper lines. (**a**) Discriminating saturation curve of 47 core SNPs. (**b**) Comparison of 47 core SNPs with 425 perfect SNPs. The horizontal axis and vertical axis denote the percentage of SNPs contributing to different genotype combinations and the number of different genotype combinations between 2 and 496 pepper lines, respectively. The left and right vertical axes correspond to the 47 core SNPs and 425 perfect SNPs, respectively. (**c**) Neighbor-joining tree of the 496 pepper lines based on the 47 core SNPs.

The fraction of informative SNPs that can differentiate the genotypes of all materials are typically selected as the core-SNP set, which is commonly used in variety and kinship identification. We found that 47 SNPs could accurately differentiate genotypes among the 484 *C. annuum* lines with a 100% accuracy rate (Figure 4a; Table S6). These 47 SNPs comprised the core-SNP set, which guaranteed that at least two distinct SNPs were present in 83% of the lines (Figure 4b). In addition, the neighbor-joining tree constructed using the 47 core SNPs clearly showed separation of the four distinct clusters (Figure 4c). Consequently, these 47 core SNPs were highly consistent with the analysis using the entire set of 425 perfect SNPs, demonstrating their suitability to effectively represent the genetic diversity of the 484 C. annuum lines, serving as reliable markers for similar varieties’ identification.

### 3.5. Genetic Variation in Pepper Populations

The analysis of molecular variance (AMOVA) for the population structure of the 484 *C. annuum lines* showed that population differences contributed to a minimum variation of 1.53%, whereas 9.32% of the total variation was attributed to differences between subpopulations (Table 1). The largest variation (86.6%) was observed within pepper lines in the subpopulations, whereas the contribution of variation within pepper lines accounted for only 2.55% of the total variation (Table 1). Concurrently, pairwise F statistic (Fst) estimates between populations and subpopulations (Table 2) revealed a high level of differentiation between POP1 and POP2 (Fst = 0.3027). The lowest pairwise Fst value (0.1672) among the four subpopulations was observed between POP2B (consisting of linear fruit lines) and POP2A (mainly composed of narrow-horn fruit lines), indicating relatively low genetic differentiation between these subpopulations. In contrast, the highest pairwise Fst value of 0.6229 was observed between POP1A (mostly comprising blocky fruit lines) and POP2B, indicating a high level of genetic differentiation.

### 3.6. Genetic Diversity and Core Pepper Lines of Subpopulations

**Figure 5:**
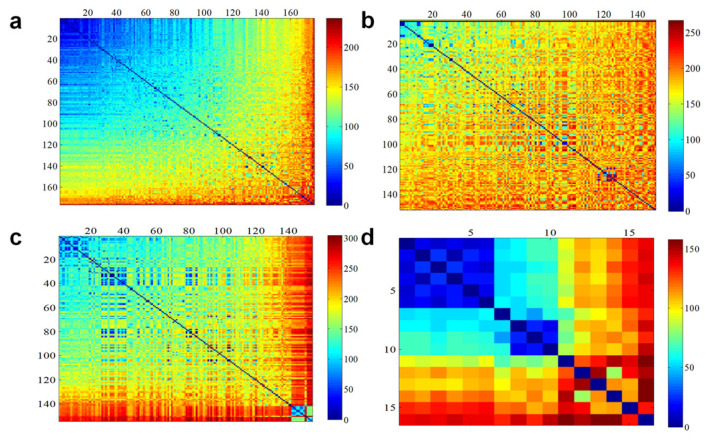
Pairwise comparison matrix heat maps of different single-nucleotide polymorphism (SNP) genotypes in subpopulations (**a**) POP1A, (**b**) POP1B, (**c**) POP2A, and (**d**) POP2B. The range from blue to red indicates an increase in the number of different SNP genotypes.

The results of the polymorphism analysis of 425 SNP markers across the four subpopulations showed that POP1A and POP1B had 420 and 425 polymorphic SNP markers, respectively, whereas all SNP markers in POP2A displayed polymorphisms (Table 3). In contrast, POP2B had the lowest number of polymorphic SNP markers, with a total of 291, which is potentially attributable to the smaller number of pepper lines in this subpopulation (Table 3). These findings underscore the distinctions among the four subpopulations. Furthermore, we compared the genetic diversity of these subpopulations characterized by fruit shape using average genetic parameters and constructed a genetic similarity matrix by calculating the number of different SNP genotypes between every pair of DNA samples (Figure 5; Table S7). POP1A had the lowest PIC (0.223), MAF (0.189), Ho (0.007), and GD (0.270) values, along with a high inbreeding coefficient, indicating lower genetic diversity in the blocky fruit lines (Table 3). By contrast, POP2A, primarily comprising narrow-horn fruit lines, displayed higher genetic diversity with elevated PIC (0.301), MAF (0.285), Ho (0.021), and GD (0.379) values, along with a lower inbreeding coefficient (Table 3). Among the 484 *C. annuum* lines, the imported inbred line “21_G28” (PI 640889) exhibited the highest genetic similarity with other pepper lines, whereas the Chinese inbred line “old_147” showed the lowest genetic similarity with other pepper lines (Table S7). For each subpopulation, the top 10% of pepper lines with the fewest differing SNP genotypes were selected as core pepper lines, resulting in a total of 49 lines (Table S8).

## 4. Discussion

High-throughput genotyping technologies are increasingly used in genetic research and breeding. In this study, based on our previous resequencing data of 35 inbred lines [32], we optimized target SNP-seq technology by identifying and expanding 425 perfect SNP markers and conducted genetic diversity analysis on 496 pepper lines. The perfect SNPs effectively distinguished the pepper lines, with the PIC values of 81.41% of the lines exceeding 0.3, and the MAF values of 47.76% of the lines exceeding 0.4. Compared to the previous study [32], we found richer variation among pepper materials, and the SNPs are more informative, making them widely applicable for pepper variety identification. It is worth noting that the Ho values analyzed in this study were lower than in previous the genetic diversity-related studies of pepper [9,11]. This indicated that the examples in our pepper lines with high homozygosity are suitable for resequencing for genome-wide association studies of important traits in the future, and the genetic polymorphism of domestic breeding resources were abundant. Furthermore, we selected a set of 47 core SNPs that could successfully distinguish between all pepper lines.

Over a prolonged period of artificial selection, five commonly cultivated species gradually emerged, with *C. annuum* being the most widely grown species at present. There has been substantial genetic research on pepper species, which has mainly focused on exploring their genetic relationships and clustering using molecular markers, whole-genome sequencing, and genotyping by sequencing [17,43,44,45,46,47,48,49,50,51,52,53]. Most of these findings suggest that *C. frutescens* is most closely related to *C. annuum*, followed by *C. chinense* [45,48,50,53]. In addition, some studies demonstrated that *C. chinense* is closely related to *C. annuum* [16,51,52]. Another study suggested that *C. frutescens* and *C. chinense* are more closely related to *C. baccatum* than to *C. annuum*, which was hypothesized to result from the geographical locations of the respective origin centers for different cultivated species [49]. The origin center of *C. baccatum* is in Bolivia, which is in close proximity to the Amazon basin, the origin site for *C. frutescens* and *C. chinense* [54,55]. Therefore, this discrepancy among studies may be attributed to the impact of artificial selection during the domestication of diverse pepper varieties in various regions and their adaptation to a wide range of global agricultural climates. Intriguingly, all of the aforementioned studies indicate that *C. frutescens* and *C. chinense* are the closest relatives, potentially because they share a common origin. The present genetic analysis of 496 pepper lines representing five pepper species revealed that *C. frutescens* was most closely related to *C. annuum*, with *C. baccatum* as the most distant species, consistent with the findings of Nicola et al. [48].

We used 484 pepper lines, including excellent pepper lines from our research team, breeding companies in China, and imported lines, to analyze the genetic diversity of *C. annuum* using phylogenetic analysis, population structure analysis, and PCA. All three methods divided the pepper lines into distinct populations according to fruit shape. In population structure analysis, 484 *C. annuum* lines were separated into two main populations, POP1 and POP2, which were each subsequently separated into two subpopulations, POP1A/POP1B and POP2A/POP2B, respectively. These four subpopulations are closely related to fruit shape, corresponding to blocky, wide-horn, narrow-horn, and linear fruits, respectively. PCA and phylogenetic analysis showed that the four subpopulations divided by fruit shape were categorized distinctly with little or no overlap, indicating that fruit shape is an important selection criterion in pepper breeding. Although our previous study [32] also identified subpopulations based on four different fruit shapes, they were divided into five subgroups, with long-horn fruit lines split into two subpopulations. Additionally, from the phylogenetic and principal component analyses of our previous study, blocky fruit lines seemed to be more closely related to long-horn fruit lines. Similarly, in a recent study [56], population structure and phylogenetic analyses of 186 pepper varieties resulted in four different fruit shape groups, with TJ and NJ seemingly representing the blocky fruit and narrow-horn fruit subpopulations of our analysis, respectively, being the most closely related; whereas in our study, the lines of blocky fruit were more strongly associated with wide-horn fruit lines, which seemed more reasonable considering the regularity of fruit shape variation. These differences may be due to the different varieties and criteria used for fruit shape classification among studies.

In terms of genetic diversity parameters in different subpopulations, POP1A, which mainly consisted of blocky fruit, had the lowest MAF, PIC, Ho, and GD values and the highest inbreeding coefficient, indicating that this subpopulation is characterized by the lowest genetic diversity and genetic background, with limited genetic materials used in breeding processes. In contrast, narrow-horn fruits showed higher diversity, with the highest Ho value and the lowest inbreeding coefficient, consistent with previous findings [32,57,58]. Different from previous studies, the PIC, Ho, and MAF of POP1B, which mainly consisted of wide-horn fruit, were higher than those of narrow-horn fruits, which also showed high diversity. Therefore, fruit shape, as one of major traits in artificial selection breeding, significantly influences the genetic structure of peppers. This study provides valuable insights for future research on the population structure variation of peppers based on fruit shape.

**Figure 6:**
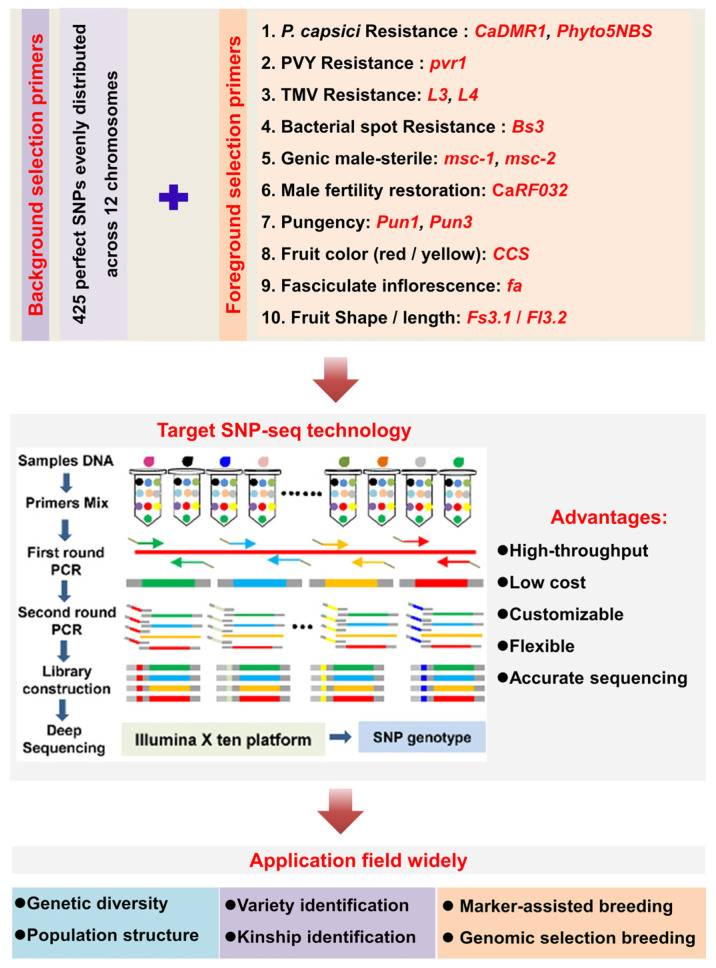
The flow chart of screening pepper materials by foreground and background markers based on optimized target SNP-seq and the application scope of this technology. References to functional sites of foreground markers are shown in Table S9.

With the continuous development of genome sequencing and resequencing technologies, as well as the assembly of large numbers of plant genomes, marker-assisted selection has become increasingly important in modern breeding practice. In our study, in addition to using perfect SNPs as background markers in target SNP-seq, we also added foreground selection markers which use specific primers to screen target genes, enabling the selection of individual plants with disease resistance and excellent traits at the seedling stage. Based on the individual disease-resistant loci added in a previous study [32,57,58], we successfully added specific primers to the flanking region of other high-quality trait-related functional sites (Table S9), such as genic male sterile genes *msc-1* and *msc-2* [59,60], capsaicin synthesis genes *Pun1* and *Pun3* [61,62], male fertility restoration gene *CaRf032* [63], functional site of fruit shape gene *Fs3.1* [51], fasciculate inflorescence gene *Cafa* [64], and fruit color (yellow/red) gene *CaCCS* [65], which were added into the perfect SNPs panel as foreground markers, combining with the background markers, and can be simultaneously detected in many pepper germplasms using target SNP-seq (Figure 6). This technology, with its advantages of low cost (in terms of both time and money) and high efficiency and flexibility, integrating foreground and background markers, can improve molecular breeding efficiency in the future and is suitable for a wide range of other applications, including genetic research and variety identification (Figure 6).

## 5. Conclusions

In this study, we analyzed the genetic diversity of 496 pepper lines from five species with target SNP-seq. The analysis of 484 *C. annuum* lines showed that their population structure was significantly influenced by fruit shape. A core set containing 47 SNPs could effectively distinguish all pepper lines, and 49 core *C. annuum* lines were identified. The core-SNP panel with important foreground markers could be an effective tool for variety identification and marker-assisted breeding.

## Figures and Tables

**Table 1:** Analysis of molecular variance results between and within populations and subpopulations.

Source of Variation	Degrees of Freedom	Sum of Squares	Mean Square	Variation Component	Percentage of Variation
Between populations	1	3612.23	3612.23	1.52	1.53
Between subpopulations within populations	2	3886.47	1943.24	9.26	9.32
Between samples within subpopulations	480	85,948.64	174.69	86.08	86.6
Within samples	484	1257.78	2.54	2.54	2.55
Total	967	94,705.12	95.57	99.4	100

**Table 2:** Pairwise F statistic (Fst) estimates among subpopulations.

(Sub) Populations	POP1	POP1B	POP2A	POP2B
POP2	0.3027			
POP1A		0.1988	0.4363	0.6229
POP1B			0.2215	0.3982
POP2A				0.1672

**Table 3:** Genetic parameters in four subpopulations.

Subpopulation	Number of Pepper Lines	Number of Polymorphic Markers	PIC	MAF	Ho	GD	Inbreeding Coefficient
POP1A	183	420	0.223	0.189	0.006	0.27	0.972
POP1B	170	424	0.312	0.319	0.007	0.4	0.981
POP2A	120	425	0.301	0.285	0.021	0.379	0.936
POP2B	11	291	0.23	0.197	0.01	0.279	0.956

Abbreviations: PIC, polymorphism information content; MAF, minimum allele frequency; Ho, observed heterozygosity; GD, genetic diversity.

## Data Availability

The original contributions presented in the study are included in the Supplementary Material, further inquiries can be directed to the corresponding authors.

## Supplementary Materials

The following supporting information can be downloaded at https://www.mdpi.com/article/10.3390/genes15020214/s1. Table S1: The species of each pepper line; Table S2: The average number of SNPs and physical distance between SNP markers on each chromosome; Table S3: Perfect SNP markers used in this study; Table S4: Genotyping of 496 pepper lines by 425 perfect SNPs; Table S5: The subpolulation and fruit shape of each pepper line; Table S6: The selected core SNPs in this study; Table S7: The number of different SNP genotypes between every pair of pepper lines in POP1A; Table S8: Core pepper lines of each subpopulations; Table S9: Foreground primers were used for the detection of the resistance loci and other tarit-realated loci through Target SNP-seq.
